# Endophthalmitis associated with Glaucoma Shunt Intraluminal Stent Exposure

**DOI:** 10.5005/jp-journals-10008-1199

**Published:** 2016-05-12

**Authors:** Hye Jin Kwon, Nathan M Kerr, Jonathan B Ruddle, Ghee Soon Ang

**Affiliations:** Resident and Clinical Observer, Department of Ophthalmology, The Royal Victorian Eye and Ear Hospital, Victoria, Australia; Glaucoma Fellow, Department of Ophthalmology, The Royal Victorian Eye and Ear Hospital, Victoria, Australia; Consultant, Department of Ophthalmology, The Royal Victorian Eye and Ear Hospital, Victoria, Australia; Consultant, Department of Ophthalmology, The Royal Victorian Eye and Ear Hospital, Victoria, Australia

**Keywords:** Case study, Endophthalmitis, Glaucoma shunt, Intraluminal stent.

## Abstract

Endophthalmitis post glaucoma drainage implant (GDI) surgery is rare, often associated with tube or plate exposure.

We report a case of endophthalmitis following glaucoma shunt intraluminal stent exposure in a patient who underwent Baerveldt glaucoma implant surgery.

Endophthalmitis following manipulation of intraluminal stents is a rare complication of GDIs but potentially vision threatening condition that needs to be carefully screened for and treated immediately.

**How to cite this article:** Kwon HJ, Kerr NM, Ruddle JB, Ang GS. Endophthalmitis associated with Glaucoma Shunt Intraluminal Stent Exposure. J Curr Glaucoma Pract 2016;10(1):36-37.

## BACKGROUND

Intraluminal stents are an effective method of reducing hypotony following glaucoma shunt surgery.^1^ We report a case of endophthalmitis associated with intraluminal stent exposure that highlights the importance of early repair of stent exposure, preferably in the operating theater.

## CASE REPORT

A 64 years old man presented to the emergency department with a 2-weeks history of a red and painful right eye. Five weeks prior to presentation, a Baerveldt 350 mm^2^ (Pharmacia and Upjohn, Kalamazoo, MI) tube with intraluminal stent (3-0 Supramid Extra TM) had been inserted superotemporally. After exiting the plate, the suture was buried in inferotemporal subconjunctival space. His glaucoma was due to a combination of neovascular and steroid-induced glaucoma for 7 months post intravitreal triamcinolone for bevacizumab-resistant diabetic macular edema. Prior to shunt surgery, the visual acuity was 6/15 in his right eye and the intraocular pressure (IOP) was 30 mm Hg on maximum medical therapy. He had a previous history of cataract surgery but no other glaucoma surgery. Surgery was uneventful and his IOP was initially well-controlled at 15 mm Hg without medical therapy.

On slit-lamp examination, trace cells were observed in the anterior chamber and the end of the intraluminal suture tip was noted to be exposed through a small conjunctival defect. The exposed suture was trimmed and the patient was prescribed topical chloramphenicol 0.5% four times a day and prednisolone acetate 1% 2-hourly.

On review 1 month later, the intraluminal suture tip was noted to be exposed again without any signs of intraocular inflammation. His right eye IOP was 22 mm Hg, persistently above target. The intraluminal suture was removed at the slit lamp with povidone iodine 5% antisepsis. Intraocular pressure was measured 30 minutes following removal and remained 22 mm Hg. He was again prescribed topical chloramphenicol 0.5% four times a day and prednisolone acetate 1% 2-hourly.

**Fig. 1:**
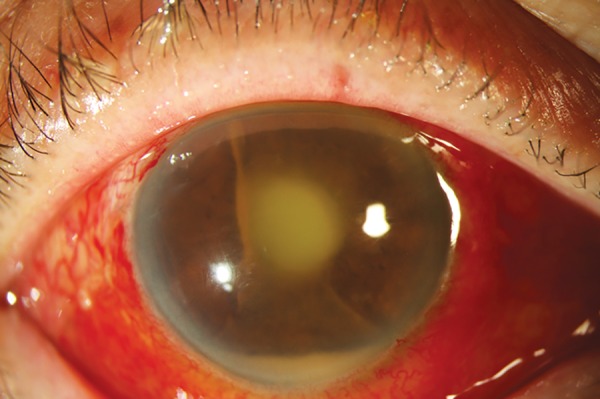
Hypopyon and track of pus from tube at presentation with endophthalmitis

One week after stent removal, the patient presented to the emergency department with a 3-day history of a red and painful right eye with reduced vision. He was using topical prednisolone acetate 1% 2-hourly but was non-adherent with topical antibiotics. On examination, the visual acuity in his right eye was perception of light. Fibrin was present in the anterior chamber and there was a 1 mm hypopyon (Fig. 1). B-scan showed vitreous debris. The patient was diagnosed with endophthalmitis and was admitted for urgent intravitreal antibiotics and vitrectomy. Repeat intravitreal tap and injections were performed on days 2 and 6 following vitrectomy due to ongoing intraocular inflammation. Microscopy showed a moderate amount of polymorphs in the aqueous and vitreous but no organisms were identified on culture at any stage. After discussion with the vitreoretinal surgeon, a decision was made not to remove the Baerveldt tube given the poor visual prognosis and because the patient’s pain and inflammation were improving. Seven months following endophthalmitis his visual acuity was hand movements and the IOP was 3 mm Hg in his right eye.

## DISCUSSION

Early hypotony may complicate glaucoma shunt surgery. Hypotony can be limited by external ligation and partial occlusion of the tube with an intraluminal stent, such as 3-0 braided nylon (Supramid Extra TM) or 3-0 polypropylene (Prolene TM).^2^ Intraluminal stents are placed in the inferior subconjunctival space to allow easy access for later removal. The intraluminal suture can be removed through a conjunctival incision in clinic or theater when sufficient bleb resistance has formed and the IOP is above target.^1^

Infective endophthalmitis has been described due to tube or plate exposure, plate revision, and conjunctival dehiscence following glaucoma shunt insertion.^3^ One study in 1990 by Ball and Scharfenberg described the development of non-infective hypopyon in 7 out of 27 eyes following removal of intraluminal stents (4-0 chromic suture, ethicone) after Molteno tube surgery. The authors postulated that this was due to a drop in IOP at the time of suture removal causing inflammatory cells located around the chromic suture to be drawn into the anterior chamber.^4^ Recent studies looking at complications of glaucoma shunts have not reported any endophthalmitis related to the intraluminal stent.^1,5^

In our case, previous exposure of the intraluminal suture may have led to colonization of the stent with microorganisms. Seeded inflammatory cells or bacteria may have subsequently tracked along the tube when the suture was removed. Despite the use of iodine antisepsis, suture manipulation in clinic may have contributed to the development of endophthalmitis. In addition, systemic and local immunosuppression from poorly controlled diabetes and intravitreal steroid injection respectively may have increased the risk of infection.^6^

## CONCLUSION

This case highlights the importance of immediate repair or removal of exposed intraluminal stents to prevent the development of endophthalmitis. Despite the burden of increased healthcare costs, stent removal in theater should be considered.^3^ Doing so enables a more controlled release of the suture in a sterile environment, thus preventing the reflux of cells into the anterior chamber and reducing the risk of intraocular infection.
